# NICU discharge preparation and transition planning: editorial

**DOI:** 10.1038/s41372-022-01310-y

**Published:** 2022-02-14

**Authors:** Betty R. Vohr

**Affiliations:** Women & Infants Hospital, Providence, RI USA

## The importance of enhanced NICU transition and discharge guidelines

Approximately 10% of newborns in the United States require care in a neonatal intensive care unit (NICU) [1]. There has been increasing evidence of the importance of comprehensive NICU care that incorporates enhanced transition and discharge guidelines as a key component of family-centered NICU care [2–4]. The need is based on the fact that many NICU graduates have special healthcare needs, utilize increased post-discharge medical and social service resources, and are at increased risk of post-discharge emergency room visits and hospitalizations [2, 5, 6]. In addition, a significant percentage of parents of NICU graduates have increased social, environmental, economic, and mental health challenges and experience the effects of disparities that impact discharge readiness and affect parenting [7–11]. Therefore, a model of multidisciplinary, family-centered care, which meets the discharge needs of the parent-infant dyad, is needed to optimize outcomes [12]. This model of discharge planning and care should begin pre-discharge (i.e., early in the NICU course), continue during the transition process, and extend to the post-discharge period. The goal is to provide a continuum of individualized, family-centered, and culturally competent support and education services that links the family to needed community resources. With the publication of this supplement, the National Perinatal Association provides a state-of-the-art framework for achieving interdisciplinary guidelines and recommendations for NICU Discharge Preparation and Transition Planning.

The following points are made: to accomplish this model of care, the NICU team needs to include not only the physicians, nurse practitioners, and nurses but parents [13–15], social workers, mental health providers, medical interpreters, and parent resource specialists [12]. An important first step is developing a trusting relationship with the family. This starts with including parents as active participants in daily rounds. Social workers also play a key role by meeting with the family soon after admission and identifying the family’s strengths and challenges, including addressing any healthcare disparities and psychosocial challenges that may impact the parent’s ability to meet the needs of their infant and their capacity to provide for those needs. For example, the development of protocols to address healthcare disparities (e.g., having public insurance or no insurance, experiencing housing instability or food insecurity) and psychosocial risks (e.g., intimate partner violence, diagnosed or undiagnosed mental health disorders, acute stress, anxiety, depression, incarceration, involvement with child protective services, limited English proficiency, and low health literacy level) are needed.

The development of a teaching manual and resource guide that provides guidance on creating a safe sleep environment, instruction for preparing feeds, tools for preventing and treating infections, information on applying for the Supplemental Nutrition Assistance Program and the Women, Infants and Children special program, clear guidance on the timing, safety, and necessity of immunizations; and instruction for safely administering prescribed and over-the-counter medications is essential to facilitating the discharge education and transition planning process.

It is important that this family resource be reviewed and revised with caregivers during their NICU stay. Assisting caregivers as they prepare for discharge is an important component of the discharge planning process and has been shown to be beneficial. Caregivers should be given referrals for support services, medical benefits assistance, utility assistance, housing resources, and community resources for mental health support while in the NICU, as this will help facilitate a smoother transition home. Findings on components of the family needs assessment can be utilized for supportive anticipatory guidance.

Discharge planning includes confirmation that the family has appointments with their primary care provider, medical specialists, home healthcare (i.e., block nursing and/or standard visiting nurse), home equipment services or durable medical equipment providers. They should be transitioned to a NICU developmental follow-up program and receive a referral for early intervention programs offered under the Individuals with Disabilities Education Act Part C. These arrangements should be documented and shared in hard copy (on paper) and/or electronically with the family. A phone call to the family within 48 h of discharge is important to confirm that all is well and, when needed, to problem solve. Continued contact with the family post-discharge in a NICU follow-up program contributes to improved family satisfaction and improved developmental and mental health outcomes. Finally, the overarching goal of enhanced transition services is to improve the care and outcomes of the parent-infant dyad and to optimize healthcare utilization. The guidelines and recommendations published in this issue by the National Perinatal Association provide specific strategies that can be implemented by NICUs to move forward in this important initiative. It is anticipated that enhanced NICU service provision will continue to grow and spread as evidence of the proposed guidelines benefits to both the family and the healthcare system are appreciated and incorporated into NICU protocols.
